# Retinal slit lamp video mosaicking

**DOI:** 10.1007/s11548-016-1377-4

**Published:** 2016-03-19

**Authors:** Sandro De Zanet, Tobias Rudolph, Rogerio Richa, Christoph Tappeiner, Raphael Sznitman

**Affiliations:** ARTORG Research Center Biomedical Engineering Research, University of Bern, Murtenstrasse 50, 3008 Bern, Switzerland; Seecrypt, Witch-Hazel Ave, Centurion, 0169 South Africa; Department of Ophthalmology, Inselspital Bern, Freiburgstrasse 12, 3010 Bern, Switzerland

**Keywords:** Slit lamp, Mosaicking, Blending, Tracking

## Abstract

**Purpose:**

To this day, the slit lamp remains the first tool used by an ophthalmologist to examine patient eyes. Imaging of the retina poses, however, a variety of problems, namely a shallow depth of focus, reflections from the optical system, a small field of view and non-uniform illumination. For ophthalmologists, the use of slit lamp images for documentation and analysis purposes, however, remains extremely challenging due to large image artifacts. For this reason, we propose an automatic retinal slit lamp video mosaicking, which enlarges the field of view and reduces amount of noise and reflections, thus enhancing image quality.

**Methods:**

Our method is composed of three parts: (i) viable content segmentation, (ii) global registration and (iii) image blending. Frame content is segmented using gradient boosting with custom pixel-wise features. Speeded-up robust features are used for finding pair-wise translations between frames with robust random sample consensus estimation and graph-based simultaneous localization and mapping for global bundle adjustment. Foreground-aware blending based on feathering merges video frames into comprehensive mosaics.

**Results:**

Foreground is segmented successfully with an area under the curve of the receiver operating characteristic curve of 0.9557. Mosaicking results and state-of-the-art methods were compared and rated by ophthalmologists showing a strong preference for a large field of view provided by our method.

**Conclusions:**

The proposed method for global registration of retinal slit lamp images of the retina into comprehensive mosaics improves over state-of-the-art methods and is preferred qualitatively.

## Introduction

To this day, the slit lamp remains the most ubiquitous microscope operated by ophthalmologists to diagnose and treat the anterior and posterior segment of the eye. It serves as the first point of contact for patients with vision problems, as one can inspect the retina using an appropriate Volk lens to compensate for the refraction of cornea and lens.

Inspecting the retina with the slit lamp, however, is a challenging task. Primarily, only a narrow slit is illuminated to reduce phototoxicity and discomfort to patients. This impedes a complete view of the retina and makes diagnosis more difficult. The refractive properties of the eye must be counteracted with an additional lens held or fixed in front of the patient’s eye. This lens can be in contact with the cornea or held at a distance from the eye, effectively trading-off better image quality for greater patient comfort. In this context, contact lenses are almost exclusively used for laser treatment, leaving routine diagnoses to be achieved with lower image quality. In such cases, reflections and misalignments in the optical path lead to a smaller field of view and image distortions. Additionally, depth of field is shallow due to large magnification, giving way to unfocused images. Owing to these reasons, video acquisition for photodocumentation is still not the common practice in clinical practice.

**Fig. 1:**
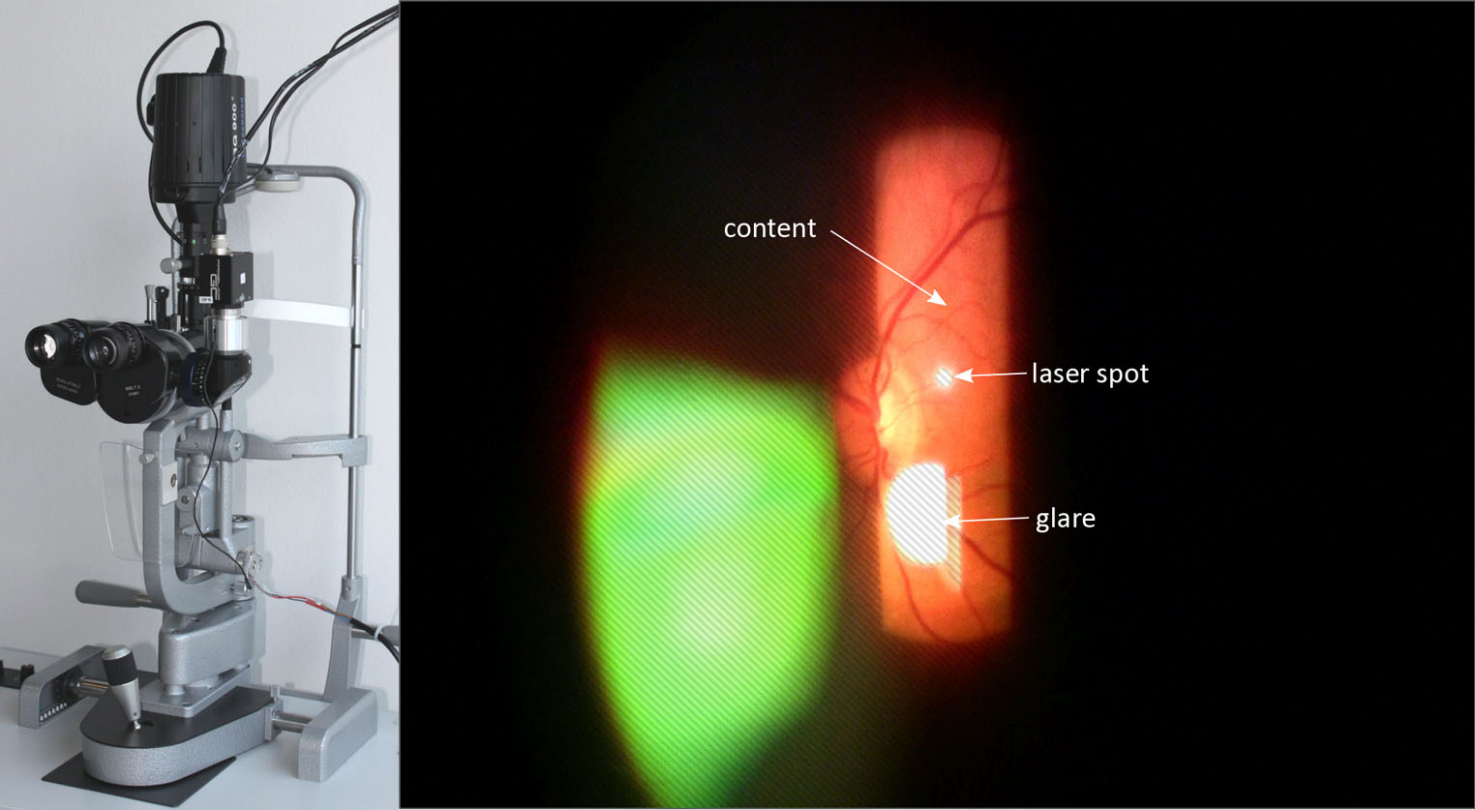
*Left* Slit lamp used in the clinic with camera mounted on a beam splitter on its right side. *Right* Rare, good quality slit lamp image of the retina. The slit is non-uniformly illuminated; especially on the borders luminosity drops off. Additionally, glare and possibly treatment laser spots occlude parts of the content

In this light, the capacity to blend a video sequence into a comprehensive retinal fundus image without artifacts greatly increases the value of slit lamp images. By simply augmenting the slit lamp with a beam splitter, a camera adapter and camera, FOV fundus photography can be attained without having to buy expensive machinery only useful for fundus photography.

A number of mosaicking methods for other microscope and endoscope image modalities have been suggested in the past. These include that of Hu et al. [5] which gives way to super-resolution. Loewke et al. [6] and Vercauteren et al. [12, 13] use distortion compensation and global alignment methods for mosaicking. Hirvonen et al. [4] use a direct affine method with frame-to-frame constraints and a Levenberg–Marquardt minimization. In the context of slit lamps, mosaicking has been demonstrated by Asmuth et al. [1]. Here they segment the slit by means of color information and shape constraints. By minimizing the sum of squared differences (SSD) between subsequent frames they find the correct translation and blend the frames with Laplacian pyramids. Alternatively, Richa et al. [9] used features to track and mosaic simultaneously with the sum of conditional variance (SCV) on videos of the retina acquired using a contact glass for the purpose of laser treatment. In the same setup, they further describe a method for online tracking and mosaicking which brings improvements over their previous work by leveraging a local illumination compensation model [8]. Their method computes a mosaic online and is able to regain tracking after losses. Both methods, however, lack a global adjustment which leads to drift and misalignments. In addition, segmentation of the slit is based on hard-set thresholds and morphological operators, which fails in more challenging imaging scenarios. It remains the case that significantly lower image quality and smaller fields of view in slit lamp images make direct registration methods impracticable.

To overcome these limitations, we present in this paper a robust method that allows for reliable slit segmentation, tracking and avoids drift to produce images useful to the clinician. Our method relies on three main steps: content extraction, feature-based mosaicking and content-aware blending. In our method, we approach the segmentation of the illuminated slit using a machine learning approach in order to deal with large glare regions and other artifacts. Content is learned on a manually annotated database. Mosaicking is then based on SURF feature matching using all possible matches between frames to allow for minute and large erroneous motion estimates in a global bundle adjustment using graph-based SLAM. Finally, registered slits are blended based on content knowledge and by weighing illumination values (feathering), counteracting diminished illumination toward the borders of slits.

We validated our method on a set of human patient videos and show the value of photodocumentation for clinicians. We demonstrated improved image quality of diagnosis by producing larger and clearer mosaics than existing methods. In addition, thanks to an improved foreground segmentation, video acquisitions of contact-free lenses with more reflections can nevertheless be used for mosaicking.

The remainder of this paper is organized as follows: In the following section, our segmentation and mosaicking method is described. In “Experiments and results” section, we present and discuss the results of experiments conducted on patients. Finally, in “Conclusion” section we conclude this work.

## Materials and methods

Our proposed method is divided into three parts: frame-wise content segmentation of the illuminated slit, inter-frame registration with bundle adjustment and content-aware blending of all the frames into a single mosaic.

### Setup

The slit lamp, as depicted in Fig. 1, was used to acquire all videos used in this work. Because this article’s main focus is on the selection of the correct region of interest, the optical setup is only discussed briefly. For this study, a slit lamp BQ 900^®^ (Haag-Streit AG, Köniz, Switzerland) equipped with a 70:30 beam splitter (70 % of light to the camera) was used. Video recording using a Prosilica GC1350c (Allied Vision Technologies GmbH, Stadtroda, Germany) was performed, generating color images of $1360 \times 1024$ pixel resolution at 20 frames per second. The camera is connected to a video-processing PC via Gigabit Ethernet. A set of 6 videos was acquired with the goal of recording as much of the retina as possible by moving the slit to anatomically important landmarks such as the optic disk and the macula.

### Segmentation

Estimation of foreground pixels is critical to create a useful mosaic due to large regions of non-informative content regions. Slit lamp images suffer from a wide variety of deteriorations, such as reflections on the lens, varying illumination and lack of focus. Being only illuminated by a narrow, vertical slit, the majority of the image does not convey any useful information. An example of a good quality slit lamp image can be found in Fig. 1. It is therefore important to automatically segment only the relevant foreground information. In the work of Richa et al. [8], the use of hard thresholding on combination of red and green channels, as well as morphological operators, is used to create a binary foreground mask. More specifically, a pixel is deemed foreground if $r - 0.7\,g > 0$, where *r* and *g* are the red and green channel pixels, respectively.

While this method is applicable to high-quality videos, where a contact lens is used, as we will show in our experiments, it fails on images acquired with contact-free lenses. This is mainly the case because pixel thresholding for content segmentation is ineffective in situations with large amounts of glare, blurriness and motion artifacts in free-hand setups.

**Fig. 2:**
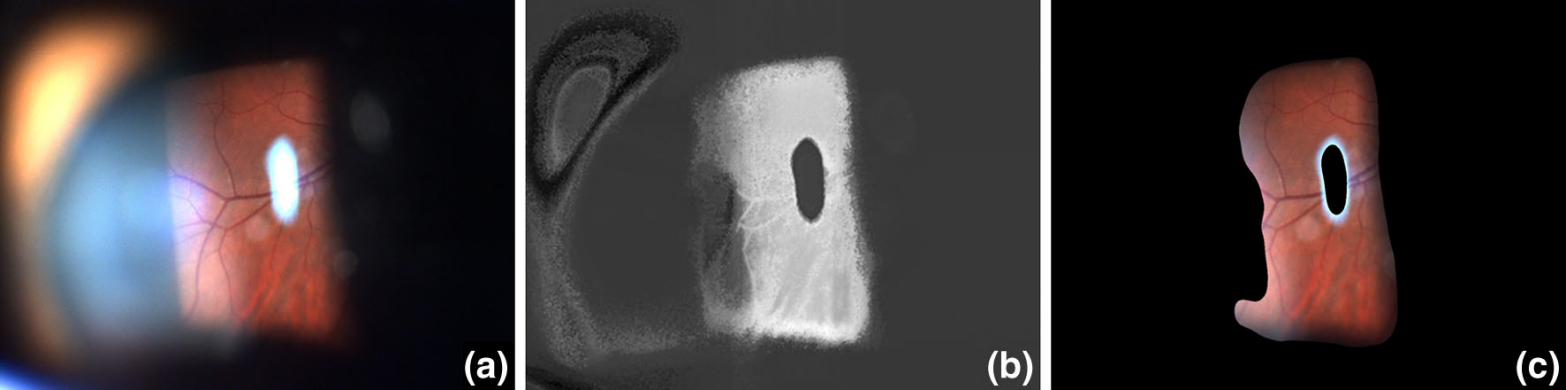
An example of the masking process. **a** Original image, **b** predicted foreground votes, **c** masked image using thresholded, smoothed and upscaled foreground votes. Due to smoothing narrow glare borders are retained

To address the more difficult cases, our approach makes use of machine learning to estimate appropriate foreground regions. We use gradient boosting as described in [2, 11] to create boosted trees for classification on a per-pixel basis (Fig. 2). To be able to compute a large number of frames in a reasonable amount of time, we opted for computationally non-demanding features, by transforming the images into different colors paces: hue, saturation and value (HSV) and luminosity and color-opponent dimensions (Lab). Using the fact that the slit appears mostly in the center of the image, we included *x* and *y* positions as potential features for the classifier to leverage. Finally, as a more computationally expensive feature, we used Gabor filters [3] under the motivation that content pixels will generate a stronger response than non-illuminated areas and glares. All images were downscaled by a factor of 4, in order to minimize noise artifacts but also to speed up training and classification times.

Our training set consisted of 283 randomly chosen frames from a set of clinical slit lamp videos. These images were manually binarized into fore- and background regions. Evaluation was performed on the full dataset with 10 % of the data used for testing and 90 % used for training, in a K-fold cross-validation fashion (see “Experiments and results” section).

### Tracking and bundle adjustment

Movement of the slit over the retina was tracked using a combination of feature descriptors and global bundle adjustment. First, movement was tracked between frames by computing a transformation between each pair of frames. The transformation is assumed to be a translation in *x* and *y* directions, while rotation is assumed to be minimal and was hence not modeled. Translation estimation is based on feature detection and matching. We used SURF for the feature point detector and descriptor, where the rotation information was ignored (upright SURF), again under the assumption of minimal rotations. The threshold for the feature retention was set to be low in order to generate a large number of points of interest. Doing so ensured that enough points are retained for a reliable transformation to be modeled.

To increase robustness, RANSAC was used to estimate the model transformation (100 iterations with an error margin of 3 px). Due to few or missing (no frame overlap) correspondences, some inter-frame transformations can not and should not be computed to create an accurate transformation result. Only frames with a minimal number of 6 correspondences were considered for the final mosaic. All other frame pairs are by consequence either not overlapping or of bad image quality.

For each frame pair, the transformation estimation is bundled into a linear system of equations according to the graph-based SLAM method described in [7]. This system can be solved efficiently offline for the position of all frames. For each translation estimation, two constraints (one for the *x*- and one for *y*-direction) are added to the linear system of equations *A*. For each translation $\Delta x$ between frame *i* and *j*, a constraint is added:

$$\begin{aligned} A_{i,i}\leftarrow 1,\quad A_{i,j}\leftarrow -1 \end{aligned}$$

and

$$\begin{aligned} b_i \leftarrow \Delta x \end{aligned}$$

for both *x* and *y* coordinates, and corresponding to the equation $x_i - x_j = \Delta x$. The system can then be expressed in the form of $Ax = b$, where *x* is the flattened vector of all mosaic positions for each frame.

The translation assumption was used to create a strong constraint on feature matching between frames, thus allowing for robust estimations. While rotations do occur around the optical axis, they are generally negligible. A similar argument can also be made of perspective distortions, which we do not account for here but which do occur in the far periphery.

### Blending

Due to strong non-uniform illumination, satisfactory blending poses a challenging problem in this application. By simply superimposing images, seams remain clearly visible where illumination differs significantly. Averaging of pixel intensities leads to blurring and a generally darker images, as more than half of the slit lamp images are not illuminated. Median filtering can be used to remove intensity outliers, for instance in case of small reflections, but this method, however, still does not account for illumination differences. In this paper, we suggest using feathering [10] in combination with foreground segmentation.

Feathering consists of computing a distance map per image and using the distance value to compute the weighted mean for the final pixel intensity, as follows

$$\begin{aligned} I_\mathrm{m}[p] = \sum \limits _i w_i[p] \cdot t_i(I_i)[p], \end{aligned}$$

where the mosaic is $I_\mathrm{m}$, the weights computed from the distance map are $w_i$, and $t_i$ is the transformation of the image $I_i$ into the mosaic. Traditionally, the map is computed on the whole image. In the case of the slit lamp, this would lead to subpar results due to large non-illuminated areas outside of the slit. Instead, the foreground segmentation is used as a mask to compute the distance map. This method has two main advantages: First, illumination intensity generally diminishes toward the border. Thanks to the distance map, these pixels take a smaller role in the mosaic and are outvoted by well-illuminated frames in the same position. On the other hand, the influence of small, wrongly segmented patches is diminished, as distance values can not become large and can again be outvoted by real content.

## Experiments and results

In order to evaluate the performance of our strategy, we evaluated both the foreground segmentation method and the overall mosaic forming strategy. Segmentation was tested on a manually annotated image dataset, while the mosaic quality was compared to state-of-the-art methods and rated by ophthalmologists.

### Segmentation

The pixel-classification model was trained on a set of 283 manually segmented video frames, where the segmentation consists of a binary foreground mask. Evaluation was performed on the full set with 10 % for testing and 90 % for training over 10 partitions, following a tenfold cross-validation scheme. Gradient boosting ran over 200 iterations with a shrinkage factor of 0.1, a subsampling factor of 0.5 and a maximal tree depth of 3. A squared loss function was used for the optimization. These parameters were evaluated to be stable for the task at hand.

**Fig. 3:**
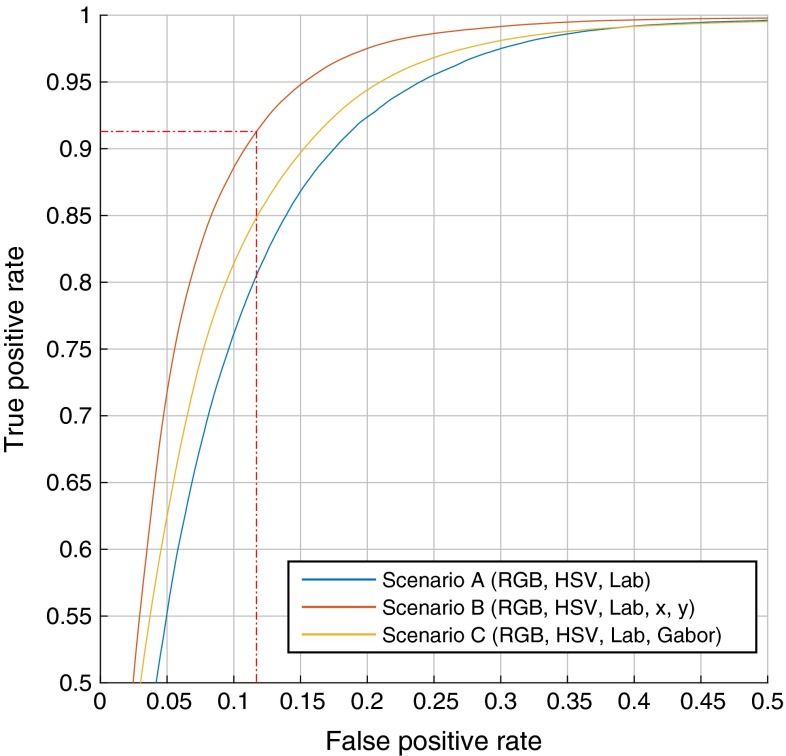
Comparison of three feature scenarios *A* (RGB, HSV, Lab), *B* (RGB, HSV, Lab, *x*, *y*), *C* (RGB, HSV, Lab, Gabor filters) in an ROC curve. Scenario B clearly shows the best performance regarding the AUC. The point of optimal operation is indicated by the *dotted red line*

**Table 1:** Comparison of foreground segmentation performance between our method and the method of Richa et al. [8]

Method	Precision	Accuracy	Sensitivity	Specificity
Richa et al. [8]	0.2	0.6	0.88	0.55
Proposed	0.93	0.96	0.91	0.98

**Fig. 4:**
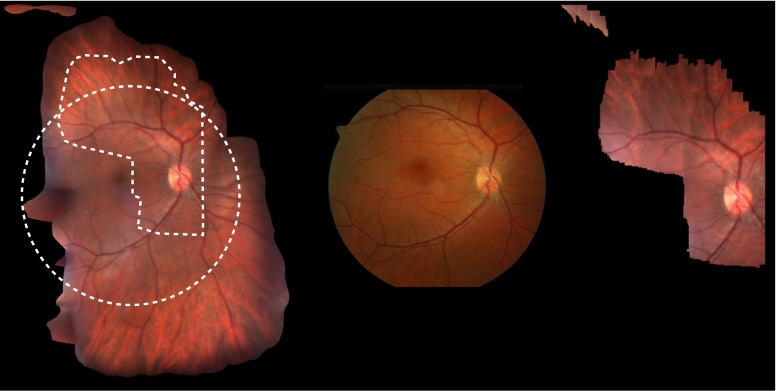
*Left* Full mosaic of slit lamp sequence of 1000 frames width a large field of view. The area covered by the separately acquired fundus photography (*center*) on the mosaic can be seen as a *white circle*. On the *right* an example of the algorithm by Richa et al. is depicted showing the maximal extend of mosaic that can be achieved in this video sequence. Its area covered is delineated in the mosaic

As mentioned in “Materials and methods” section, the following feature set scenarios were considered:

- Model A: RGB, LAB, HSV
- Model B: RGB, LAB, HSV, x, y
- Model C: RGB, LAB, HSV, Gabor Filter Bank

Model A considers different color spaces, model B adds position information, and model C, instead, uses Gabor filters [3]. For the Gabor filter bank, we used 3 scales and 4 directions. The 10 models from cross-validation were evaluated using the AUC of the mean ROC curve. In Fig. 3, the mean ROC curves of the three scenarios are compared. With an AUC of the ROC curve of 0.9557, scenario B presents itself as the best choice for foreground segmentation above scenario A (AUC = 0.9293) and C (AUC = 0.9405). Evidently, spatial information has a big influence on the outcome of the segmentation. This can be explained by the fact that slits almost exclusively occur in the center of the image, which reduces the search space significantly.

As a baseline, the method proposed by Richa et al. [8] was compared. Our method performs with a higher specificity, sensitivity, precision and accuracy at the optimal point of operation, as shown in Table 1.

**Fig. 5:**
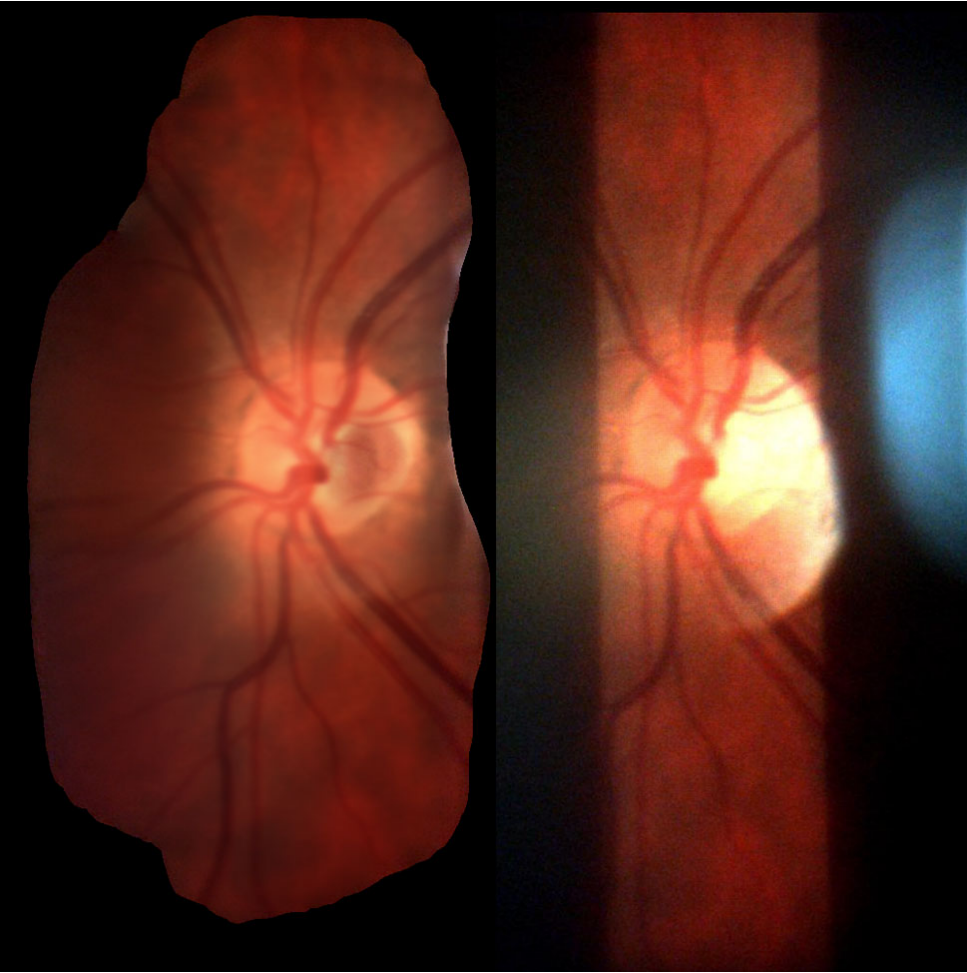
*Left* Full mosaic of slit lamp image where slit did not move. Different views are fused to reveal more detail than is available in any single frame. Additionally, noise is removed thanks to weighted averaging. On the *right* a single frame is depicted with visible noise and glare

**Fig. 6:**
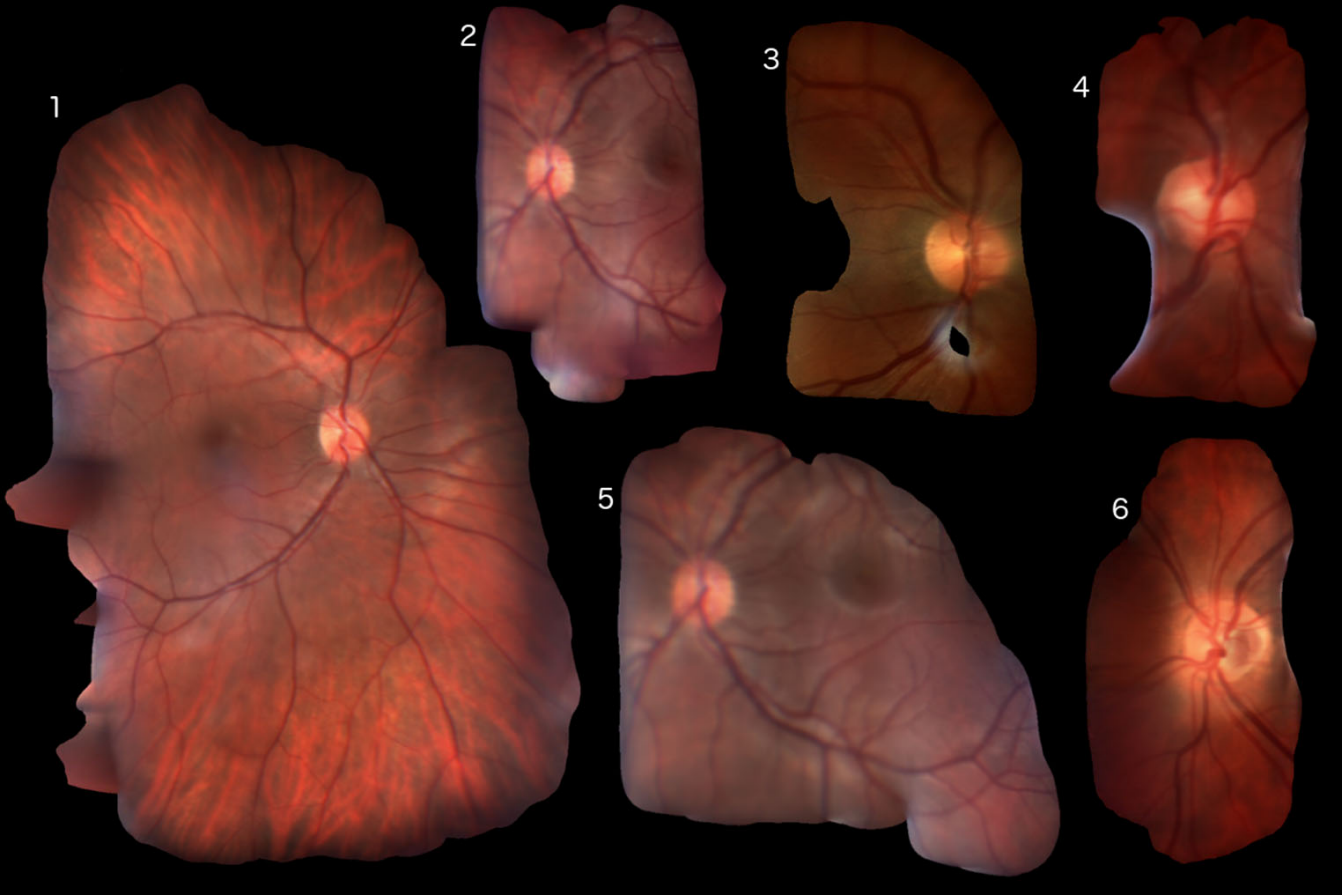
All mosaics presented to the ophthalmologists. Notice that mosaic 2 and mosaic 5 stem from the same patient, but from two different acquisitions

### Mosaic

Mosaics were generated from all 6 videos and are shown in Fig. 6. In some videos, the ophthalmologist was not able to move far from the optic disk. Even in such cases the image quality was improved, by reducing noise and removing glares (see Fig. 5). In Fig. 4, our method is compared to a corresponding fundus photography and the mosaicking result of Richa et al. It can be seen that using global bundle adjustment, a considerably larger region can be incorporated, making up for an average of 35 % increased field of view.

### Evaluation by physicians

Two clinicians were presented with mosaics generated using the technique of Richa et al. [8] and our method. These clinicians were tasked to evaluate the viability of the mosaics for clinical use (see Fig. 6). All six mosaics, generated by our method and the method of Richa et al. [8], were compared side to side in a random order. The ophthalmologists were asked to rate the mosaic they preferred for clinical use and which one had the better quality. For all six mosaics, our method was preferred for use by clinicians. Image quality was rated equally or better in all cases, but was less of a concern to the ophthalmologists. The clinicians’ preference to our mosaics was largely due to the much bigger field of view provided by global bundle adjustment. Since more area of the retina is considered, decreased image quality was less of a concern from the physicians’ point of view.

### Computation time

The computation time of our method mainly depends on the number of usable frames acquired. In general, within one minute, an ophthalmologist has time to scan over all diagnostically relevant retinal areas. Our MATLAB implementation takes less than 10 min for such a video and could further be optimized, making it acceptable for a clinician to use.

## Conclusion

In this work, we present a method to improve slit lamp acquisitions by creating global mosaics of the retina when poor quality video frames are present. Our method improves previous work in three ways. First, we propose a machine learning-based pixel-wise segmentation of the slit to remove glare, reflections and unlit areas. Second, frames are registered using SURF features and bundle adjusted to remove drift, caused by inaccurate motion estimation. Last, we propose blending by a weighted average derived from the previously generated slit mask. By doing so, our generated mosaics could be significantly enlarged by including more bundle adjusted frames. We evaluated mosaics with ophthalmologists who preferred these larger mosaics, as additional important retinal structures could be seen.

Steps to further improve mosaics are the incorporation of image sharpness into the blending scheme in order to select the best image quality for a given pixel. Since bundle adjustment only accounts for translation, slight rotations of the eye can lead to a less defined image. The incorporation of potential rotations in the bundle adjustment scheme would further improve mosaic quality as well.
